# Excessive body weight and its contributing factors in visually impaired patients in northwest Ethiopia, 2024

**DOI:** 10.3389/fendo.2025.1514308

**Published:** 2025-03-20

**Authors:** Baye Ashenef, Bayu Wondimneh Yimenu, Enatnesh Essa Osman, Derese Sinamaw, Gelagey Baye, Zigale Hibstu Teffera, Mamaru Getinet Engida, Adane Adugna, Enyew Fenta Mengistu, Temesgen Baylie, Mohammed Jemal

**Affiliations:** ^1^ Department of Biomedical Sciences, School of Medicine, Debre Markos University, Debre Markos, Ethiopia; ^2^ Department of Medical Laboratory Sciences, College of Health Sciences, Debre Markos University, Debre Markos, Ethiopia

**Keywords:** excessive body weight, visual impairment, northwest Ethiopia, overweight, obesity

## Abstract

**Background:**

Excessive body weight are global health challenge affecting individuals across all age groups. They result from an imbalance between calorie intake and expenditure. Individuals with visual impairment are particularly vulnerable due to reduced physical activity, leading to higher rates of excessive body weight. This study assessed the prevalence and determinants of excessive body weight among visually impaired patients in northwest Ethiopia in 2024.

**Method:**

A multi-centered institution-based cross-sectional study was done with 384 visually impaired patients selected using a simple random sample technique. An interviewer-administered structured questionnaire and physical assessments were used to gather data. The crude and adjusted odds ratios, as well as the 95% confidence intervals, were calculated. Bivariate and multivariate logistic regression analyses were performed. Variables having a p-value < 0.05 were considered substantially associated with excessive body weight.

**Results:**

The prevalence of excessive body weight was 27.9% (95% CI: 23.2–32.8). Factors significantly associated with excessive body weight included being aged 65 or older (AOR = 5.43, 95% CI: 1.22–24.1), urban residency (AOR = 4.84, 95% CI: 2.95–7.95), and having visual impairment for five or more years (AOR = 3.33, 95% CI: 1.88–5.89).

**Conclusion:**

Excessive body weight affects nearly one-third of visually impaired patients in northwest Ethiopia, with significant associations found for older age, urban residence, and long-term visual impairment. Integrating weight management programs, physical activity promotion, nutritional education, and community support is essential to improve health outcomes. Future studies using prospective cohort designs are recommended to explore causal relationships.

## Introduction

Visual impairments often lead to reduced physical activity and difficulty managing food (1), contributing to unhealthy eating habits (2). Additionally, psychological issues such as depression, anxiety, and chronic stress can trigger hormonal changes and emotional eating, increasing their risk of excessive body weight (3)

Excessive body weight, defined by a BMI ≥ 25 kg/m² (4), encompasses both overweight and obesity and is characterized by an abnormal accumulation of body fat, significantly increasing health risks for individuals of all age groups (5–8). Excessive body weight arises mainly from an energy imbalance between calorie intake and expenditure (9). Contributing factors include reduced physical activity (10), insufficient sleep (11), abnormal physiological conditions (hypothyroidism, Cushing’s syndrome, growth hormone deficiency, eating disorders) (12–14), medications (like insulin, steroids, sulfonylureas, antidepressants, and hormonal contraceptives) (15), Genetic predisposition (14), and late-age pregnancy (16, 17).

These conditions are major risk factors for a range of non-communicable diseases, including cardiovascular diseases (heart disease and stroke), type 2 diabetes, musculoskeletal disorders like osteoarthritis, and certain cancers (e.g., endometrial, breast, and colon cancers) (18–20).

Globally, the prevalence of excessive body weight is rising. In 2022, the WHO reported that 2.5 billion adults were overweight, and 890 million were obese, with this trend evident in both developing and developed countries (16, 19). In Africa, overweight and obesity prevalence among adults ranges from 13.6% to 31%, while among children and adolescents, it ranges from 5% to 16.5% (16, 21). In Ethiopia, the prevalence of overweight and obesity among adults was reported at 20.4% and 5.4%, respectively (22).

Among adults with visual impairments, the prevalence of excessive body weight varies significantly by country, 22.2% in Korea (23), 36% in the United States (24), and 38% in India (25). However, no studies have investigated the prevalence of excessive body weight among visually impaired adults in Ethiopia.

Excessive body weight significantly affects individuals with visual impairments and their families (26). These conditions can reduce the quality of life (QOL) (27), and contribute to physical inactivity, resulting in low socioeconomic status (28). They also increase the need for self-care assistance, adversely affecting mental health, social functioning, employment, and educational opportunities (27, 29).

Evidence indicates that excessive body weight is associated with various factors including family history (30), age (31), sex (32), income (33), educational attainment (34), dietary type (35), length of sleep in a day (36), cigarette smoking (37), and alcohol consumption (38), khat chewing (39).

Despite these associations, no studies have explored the prevalence and risk factors of excessive body weight among visually impaired adults in Northwest Ethiopia. This study aims to fill that gap by investigating the prevalence and determinants of excessive body weight among visually impaired individuals attending follow-up visits in hospitals in Northwest Ethiopia. The findings emphasize the need to address excessive body weight within this population and implement targeted preventative strategies.

## Methods and materials

### Study setting and period

This study was conducted in hospitals located in Northwest Ethiopia from February 1 to March 30, 2024. These hospitals provide comprehensive care and support services to a population exceeding 25 million, including approximately 5,000 visually impaired patients who receive follow-ups and comprehensive healthcare services (unpublished data).

### Study design and population

An institutional-based cross-sectional study was conducted among visually impaired patients attending hospitals in Northwest Ethiopia. The source population comprised all visually impaired patients on follow-up in the selected hospitals, while the study population included visually impaired patients on follow-up during the study period at randomly selected hospitals.

### Inclusion and exclusion criteria

#### Inclusion criteria

- Adults aged 18 years and above with documented visual impairment.
- Residents of Northwest Ethiopia for at least six months.
- Individuals who provided informed consent.

#### Exclusion criteria

- Patients with severe illnesses, pregnancy, or mobility disabilities.
- Individuals with chronic conditions such as hypothyroidism, Cushing’s syndrome, or other infections that could interfere with body weight.
- Patients with severe cognitive impairments, acute psychiatric conditions, or those on weight-altering psychiatric medications.
- Individuals with hearing impairments or those taking medications affecting body weight

Sample size determination

The sample size was determined using a single population proportion formula.


$$n=\frac{{\left({\text{Z}}_{\text{α}/2}\right)}^{2}\times \text{ p}(1-\text{p})}{\;{\text{d}}^{2}}=\frac{{\left(1.96\right)}^{2}\times (0.5)(0.5)}{{\left(0.05\right)}^{2}}384$$

Assumption:

n = sample size, P = proportion of excessive body weight = 50% since the study on excessive body weight among visually impaired patients was not conducted in the study area, d = Margin of sampling error tolerated- 5% (0.05), α = Critical value at 95% confidence interval of certainty (1.96).

### Sampling procedure

A combination of simple and systematic random sampling techniques was used to select participants. Three hospitals (Finote Selam General Hospital, Debre Markos Comprehensive and Specialized Hospital, and Mertulemariam Primary Hospital) were randomly selected using the lottery method. From the ophthalmic clinic records, a sampling frame of visually impaired patients was established. Systematic random sampling was applied, with every 2^nd^ patient selected during data collection (sampling interval K=7, calculated by dividing the total number of visually impaired patients during the study period [2,500] by the sample size [384]). The starting point (patient number 2) was determined through the lottery method. A proportional number of participants were selected from each hospital to ensure representativeness.

### Study variables

#### Dependent variable

- Excessive body weight (Yes/No).

#### Independent variables

- Sociodemographic factors: Age, gender, residence, income, employment, education, marital status.
- Health-related factors: Blood pressure, family history of diabetes, diabetes mellitus
- Behavioral factors: Khat chewing, smoking, alcohol consumption, sleep duration, dietary habits, level of visual impairment

### Operational definitions

Excessive body weight: BMI ≥ 25 kg/m² (4)

Diabetes Mellitus: a random plasma glucose level of ≥200 mg/dL (40).

Visual impairment: Having presenting visual acuity less than 6/12 in the better eye (41).

Mild visual impairment: Having visual acuity worse than 6/12 but better than 6/18 in the better eye (41).

Moderate visual impairment: Presenting distance visual acuity (VA) worse than 6/18 but better than 6/60 in the better eye (42).

Severe visual impairment: Presenting distance VA worse than 6/60 but better than 3/60 in the better eye (42).

Blindness: Presenting VA worse than 3/60 (43).

Current smoker: An adult who has smoked 100 cigarettes in his or her lifetime and who currently smokes cigarettes (44).

Ever alcohol user: Use of alcohol, at least once in an individual’s lifetime (45).

Current alcohol user: A person who consumed alcohol at least once within the last 30 days (45).

Duration of sleep: a short length of sleep of ≤5 hours and long sleep of ≥9 hours (46).

### Data collection procedure and tools

Data collection occurred from February 1 to March 30, 2024, using a structured, interviewer-administered questionnaire developed from various validated sources (47, 48). The questionnaire collected data on medical assessments, substance use, and sociodemographic characteristics, while blood pressure and body mass index (BMI) measurements were taken for patients with visual impairment. Two BSc clinical nurses were recruited at each hospital to collect and document data.

### Examinations and measurements

#### Anthropometric and blood pressure measurements

Weight and Height: Participants’ weight was measured using a standard balance scale, and height was recorded with a stadiometer. BMI was calculated by dividing weight (kg) by height squared (m²). Blood Pressure: Blood pressure was measured using a calibrated sphygmomanometer, ensuring standard protocol adherence.

### Data analysis procedure

The collected data were checked for completeness and entered Epi data version 4.6. Then it was exported into SPSS version 26 for analysis. The crude and adjusted odds ratios were used to measure the association between study variables, and 95% confidence intervals were calculated. Descriptive measures such as median, interquartile range, and frequencies were calculated. Multicollinearity among selected independent variables was checked, and the variance inflation factor was found to be acceptable (less than 2) and model fitness was checked by the Hosmer and Lemeshow test at p-value >0.05 (p=0.965). Both bivariable and multivariable binary logistic regression models were used to identify associated factors of excessive body weight. Those variables having a p-value of <0.25 in the bivariable binary logistic regression analysis were selected for multivariable binary logistic regression. Those variables with a p-value of ≤ 0.05 in multivariable binary logistic regression were declared as having a statistically significant association with excessive body weight. Results were organized and presented by using frequency tables, graphs, and charts.

### Data quality management

To ensure the data quality, high prominence was given in designing the data collection instrument. The questionnaire was pre-tested in a setup having similar socio-cultural characteristics with the 20 study participants at Bichena primary hospitals before the actual study begins. It helps to check its wording and sort out language barriers and contextual variations on the structured questionnaire. Training for both data collectors and supervisors regarding the study objectives, ethical considerations, data collection tools, interview techniques, measurement techniques, and strategies to ensure data quality and consistency was given for one day. Throughout the data collection, data collectors were supervised.

## Results

### Socio-demographic, clinical, substance use, and behavioral characteristics of study participants

The study involved 384 visually impaired patients, achieving a 100% response rate. Participants ranged in age from 18 to 90 years, with a median age of 59 years (± 17) and an interquartile range (IQR) of 49–69 years. Among the participants, 225 (58.6%) were male, 243 (63.3%) were married, 203 (52.9%) resided in rural areas, and 289 (75.3%) were illiterate (**Table 1**).

**Table 1:** Sociodemographic characteristics of visually impaired adult patients in selected hospitals of northwest Ethiopia, 2024 (n=384).

Variables	Frequency	Percentage (%)
Age of participants (years)		
<40	13	3.39
40-64	167	43.49
≥65	204	53.13
Sex of participants		
Female	159	41.4
Male	225	58.6
Marital status		
Married	243	63.3
Divorced	27	7.0
Widowed	106	27.6
Single	8	2.1
Residence		
Urban	181	47.1
Rural	203	52.9
Educational level		
Unable to read and write	289	75.3
Primary	45	11.7
Secondary and preparatory	22	5.70
College and university	28	7.30
Occupation		
Farmer	150	39.10
Civil servant	16	4.20
Merchant	32	8.30
Housewife	118	30.7
Unemployed	13	3.40
Retire	55	14.3
Income (Ethiopian Birr)		
<2000	234	61
2000-5000	90	23.4
>5000	60	15.6

Of the participants, 244 (63.5%) had experienced visual impairment for less than five years. About half of the respondents (51.8%) reported sleeping between 5 and 8 hours per night. The majority, 232 (60.4%), were non-vegetarians. Additionally, 331 participants (86.2%) had consumed alcohol at some point in their lives, but only 47 (12.2%) reported drinking alcohol within the past 30 days, primarily traditional beverages such as *tela* or *tej.* Among the respondents, 63% had elevated systolic blood pressure, while 48.2% showed elevated diastolic blood pressure. Of the visually impaired respondents, 17.7% had diabetes mellitus (**Table 2**).

**Table 2:** Clinical and behavioral characteristics of visually impaired adult patients in selected hospitals of northwest Ethiopia, 2024 (n=384).

Variables	Frequency	Percentage (%)
Duration for visual impairment		
<5 years	244	63.5
≥5 years	140	36.5
Duration of sleep per day		
<5 hours	3	0.80
5-8 hours	199	51.8
≥9 hours	182	47.4
Dietary types		
Vegetarians	152	39.6
Non-vegetarians	232	60.4
Chewing chat		
Yes	1	0.3
No	383	99.7
Ever alcohol drinker		
Yes	331	86.2
No	53	13.8
Current alcohol drinker		
Yes	47	12.2
No	337	87.8
SBP (mmHg)<140	142	37.0
≥ 140	242	63.0
DBP (mmHg)< 90	199	51.8
≥ 90	185	48.2
Body mass index (kg/m^2^)		
< 18.5	11	2.90
18.5-24.99	266	69.3
25-29.99	96	25.0
≥30	11	2.90
Family history of DM		
Yes	11	2.9
No	373	97.1
Diabetes mellitus		
Yes	68	17.7
No	316	82.3

### Prevalence of excessive body weight

**Figure 1:**
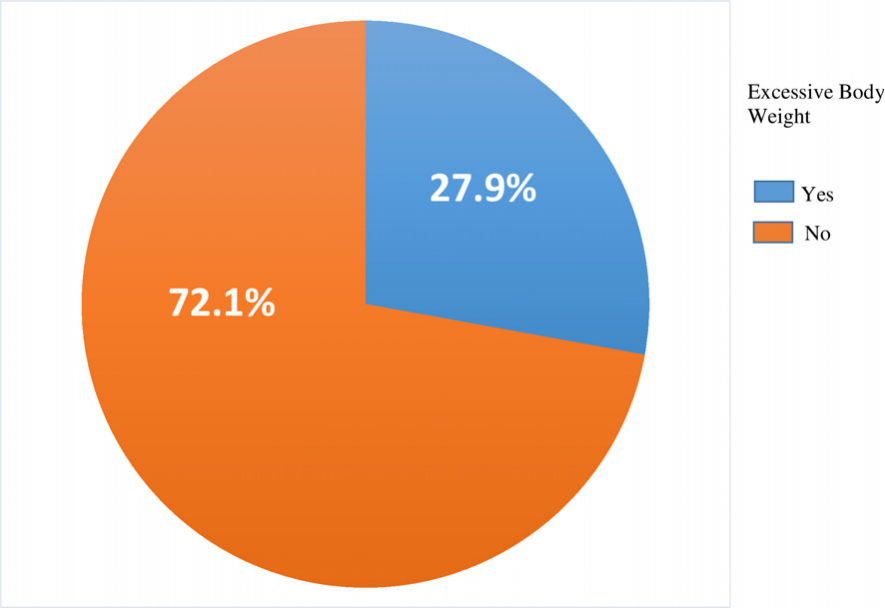
Prevalence of excessive body weight among visually impaired adult patients in selected hospitals of northwest Ethiopia, 2024 (n=384).

The overall prevalence of excessive body weight among visually impaired patients was 27.9% (95% CI: 23.2- 32.8) (**Figure 1**). Among the respondents, 13.28% with mild visual impairment, 8.6% with moderate visual impairment, and 5.99% with severe visual impairment were found to have excessive body weight (**Table 3**).

**Table 3:** Distribution of excessive body weight among visually impaired adult patients by level of visual impairment in selected hospitals of northwest Ethiopia, 2024 (n=384).

Level of visual impairment	Excessive body weight		Total (%)
	Yes (%)	No (%)	
Mild	51(13.28)	106(27.6)	157(40.89)
Moderate	33(8.6)	100(26.04)	133(34.63)
Severe	23(5.99)	71(18.49)	94(24.48)
Total	107(27.9)	277(72.1)	384(100)

### Determinant factors of diabetes mellitus among hypertensive patients

In the bivariate analysis, factors such as age, marital status, family size, residence, education level, occupation, duration of visual impairment, dietary habits, current alcohol consumption, and isolated diastolic blood pressure were associated with excessive body weight. However, the multivariate analysis, which adjusts for potential confounders, revealed that age (65 years or older), residence, and the duration of visual impairment were significantly associated with excessive body weight.

Respondents aged 65 years or older were significantly more likely to have excessive body weight, with 5.43 times higher odds compared to those under 40 years (AOR = 5.43, 95% CI: 1.22–12.1). Similarly, individuals residing in urban areas had 4.84 times higher odds of excessive body weight compared to those living in rural areas (AOR = 4.84, 95% CI: 2.95–7.95). Furthermore, individuals with visual impairment lasting five or more years were 3.33 times more likely to have excessive body weight compared to those with a shorter duration of impairment (AOR = 3.33, 95% CI: 1.88–5.89) (**Table 4**).

**Table 4:** Bivariable and multivariable binary logistic regression analysis of factors associated with excessive body weight among visually impaired adult patients in selected hospitals of northwest Ethiopia, 2024 (n=384).

Variables	Excessive body weight		AOR (95% CI)	p-value
	Yes (%)	No (%)		
Age <40	2(15.4)	11(84.6)	1.00	
40-64	37(22.16)	130(77.84)	1.9(0.34-10.75)	0.13
≥65	68(33.3)	136(66.7)	5.43(1.22-12.1)	0.001
Marital status				
Single	2(25)	6(75)	1.00	
Married	78(32.1)	165(67.9)	2.12(0.24-8.51)	0.216
Widowed	19(17.92)	87(82.08)	0.45(0.13-1.50)	0.346
Divorced	8(29.63)	19(70.37)	0.66(0.22-1.95)	0.462
Number of families				
<3	18(19.35)	75(80.65)	1.00	
3-5	72(30.25)	166(69.75)	1.7(0.66-4.38)	0.5514
>5	17(32.07)	36(67.93)	1.3(0.64-2.70)	0.527
Residence				
Urban	79(43.65)	102(56.35)	4.84(2.95-7.95)	0.003
Rural	28(13.8)	175(86.2)	1.00	
Educational level				
Unable to read and write	61(21.1)	228(78.9)	0.5(0.1-2.65)	0.247
Primary Secondary and	21(44.4)	24(55.6)	0.98(0.19-5.20)	0.413
Preparatory	12(54.55)	10(45.45)	2.13 (0.39-11.62)	0.966
College	9(56.25)	7(43.75)	2.0(0.38-11.17)	0.382
University	4(33.33)	8(66.67)	1.00	
Occupation				
Civil servant	6(37.5)	10(62.5)	1.00	
Farmer	36(24.00)	114(76.00)	5.18(0.99-17.06)	0.777
Housewife	28(23.73)	90(76.27)	2.4 (0.45-12.6)	0.200
Merchant	15(46.88)	17(53.12)	1.95 (0.36-10.15)	0522
Retire	20(36.36)	35(63.64)	1.14(0.26-5.08)	0.678
Unemployment	2(15.38)	11(84.62)	1.17(0.1-13.53)	0.829
Duration of VI				
<5 years	47(19.26)	197(80.74)		
≥5 years	60(42.86)	80(57.14)	3.33(1.88-5.89)	0.005
Dietary type				
Vegetarians	53(35.1)	98(64.9)	1.00	
Non-vegetarians	54(23.18)	179(76.82)	1.01(0.57-2.17)	0.810
Current alcohol drinking				
Yes	20(42.55)	27(57.45)	0.49 (0.22-1.08)	0.086
No	87(25.82)	250(74.18)	1.00	
Diastolic BP				
<90mmhg	51(25.63)	148(74.37)	1.00	
≥90mmhg	(5630.27)	129(69.73)	0.65(0.37-1.14)	0.166

COR, Crude odds ratio; AOR, Adjusted odds ratio; CI, Confidence interval; VI, visual impairment; DBP, diastolic blood pressure.

## Discussion

This study aimed to assess the prevalence of excessive body weight and its associated factors among visually impaired patients in hospitals in northwest Ethiopia in 2024. The prevalence of excessive body weight among these patients was found to be 27.9%, which is higher than the 22.2% reported in a Korean population-based study (23). This discrepancy might be due to this study having a smaller sample size compared to the Korean study, which included 8,435 participants. Additionally, the higher illiteracy rate among participants in this study likely contributed to lower levels of physical activity, potentially leading to an overestimation of excessive body weight prevalence. Furthermore, differences in study design may have influenced the results. While the Korean study employed a population-based prospective cohort design, this research used an institution-based cross-sectional approach. The hospital-based nature of this study might also have inflated the prevalence estimate compared to the broader, community-based Korean survey.

In contrast, the prevalence of excessive body weight in this study was lower than findings from studies conducted in the United States (36%) (24) and India (38%) (25). This discrepancy may stem from variations in participant age ranges. While both the U.S. and Indian studies focused on individuals aged 60 years and older, this study included participants aged 18 and above. Aging is often linked to decreased physical activity, resulting in reduced total energy expenditure and disturbances in energy balance, both of which can contribute to weight gain (49). Lifestyle differences between study populations may explain the observed variation. In the U.S. study, most participants were current alcohol drinkers and smokers, while in this study; the majority did not consume alcohol or smoke. Alcohol consumption, particularly binge drinking, significantly contributes to excessive body weight due to its high caloric content (7 calories per gram) (38). It reduces fat oxidation, promotes fat storage (especially abdominal), disrupts appetite-regulating hormones (leptin and ghrelin), impairs sleep, and lowers physical activity due to fatigue and hangovers (50, 51, 52–54). Smoking contributes to weight gain through metabolic dysregulation, including insulin resistance and altered fat metabolism, especially abdominal fat accumulation (55, 56). It disrupts appetite regulation via dopamine changes, increases cravings for high-calorie foods, and can lead to weight gain during cessation due to increased hunger and slower metabolism (57–61). Smoking is further linked to leptin resistance and chronic inflammation, exacerbating fat accumulation (62).

Our results revealed that 13.28%, 8.6%, and 5.99% of those with mild, moderate, and severe visual impairment respectively had excessive body weight. This discrepancy might be due to persons with severe visual impairment experiencing mobility difficulties that limit access to high-calorie diets, while reduced physical activity in mild cases may encourage weight gain (63). Additionally, individuals with advanced visual loss may adopt healthier diets due to caregiver support, while socioeconomic constraints might further reduce caloric intake (64).

Older adults (aged 65 and above) are significantly more likely to have excessive body weight, as supported by studies conducted in Ethiopia (65), Tanzania (66), Malaysia (67), and Norway (68). This association may be attributed to the close link between aging, vision impairment, and excessive body weight, which creates a cycle that negatively impacts health and quality of life in older individuals (69). Aging contributes to weight gain through a combination of metabolic, hormonal, and lifestyle changes (70). Sarcopenia, the age-related loss of muscle mass, lowers the basal metabolic rate, reducing calorie expenditure and increasing fat storage (71). Hormonal changes, such as reduced levels of growth hormone, testosterone, and estrogen, further promote fat accumulation and increase insulin resistance, exacerbating weight gain (72). Additionally, physical activity often declines with age due to mobility challenges, leading to fewer calories burned (73). Meanwhile, many older adults maintain their existing eating habits, often preferring high-calorie, low-nutrient foods, which further elevates the risk of weight gain (74).

Living in urban areas was significantly linked to excessive body weight, as observed in studies from Switzerland (75), Algeria (76), Bangladesh (77), and Ethiopia (78). Living in cities may contribute to excessive body weight due to several reasons (75). Urban dwellers often lead sedentary lifestyles, with jobs that require long periods of sitting and the use of cars or public transportation, which limits opportunities for physical activity (79). additionally, urban areas tend to have fewer safe spaces for exercise (80),, and the easy availability of unhealthy, high-calorie foods from fast food outlets and convenience stores encourages poor dietary habits (81). Collectively, these factors increase the likelihood of weight gain and make weight loss more challenging, contributing to higher rates of excessive body weight among urban populations.

A duration of visual impairment lasting five years or more is associated with excessive body weight. This could be attributed to the long-term effects of visual impairment, particularly when it persists for over five years, leading to an increase in body weight. Reduced physical activity often results from mobility issues, safety concerns, and reliance on others for assistance, which limit opportunities for exercise (82). This, in turn, fosters a more sedentary lifestyle, with individuals spending more time indoors and engaging in less physical movement. Emotional eating may also arise from stress, anxiety, or sadness linked to visual impairment, and difficulties in assessing food quantities or preparing meals can result in poor nutritional choices, typically favoring convenient or calorie-dense foods (83). These changes in physical activity and diet, combined with reduced overall energy expenditure, contribute to the risk of weight gain. Furthermore, limited access to healthcare resources and exercise programs tailored for individuals with visual impairments can make it challenging to effectively manage weight (84).

In this study, many of the questions in the data collection instrument relied on participants’ recall, which may introduce recall bias. Additionally, as a cross-sectional study, it does not allow for the establishment of a cause-and-effect relationship. Given that this study was conducted in a hospital setting, its findings on the prevalence of excessive body weight cannot be generalized to the broader community.

## Conclusions and recommendations

This study found a 27.9% prevalence of excessive body weight among visually impaired patients in northwest Ethiopia. Factors such as older age, urban residency, and long-term visual impairment were significantly associated with higher rates of excessive body weight. To manage excessive body weight, promoting physical activity, nutritional education, specialized healthcare, weight monitoring, lifestyle counseling, and emotional support are crucial. Additionally, conducting a prospective cohort study would be valuable in establishing a cause-and-effect relationship. Future studies should also ensure the inclusion of individuals with mobility disabilities. Furthermore, the causes of visual impairment and obesity-related complications in patients with visual impairment such as insulin resistance, T2DM, lipid disorders, and hyperuricemia, should be included in future studies.

## Data Availability

The datasets presented in this study can be found in online repositories. The names of the repository/repositories and accession number(s) can be found in the article/supplementary material.

## Appendix

**Table 1:** Bivariable and multivariable binary logistic regression analysis of factors associated with excessive body weight among visually impaired adult patients in selected hospitals of northwest Ethiopia, 2024 (n=384).

Variables	Excessive body weight		COR (95% CI)	AOR (95% CI)
	Yes (%)	No (%)		
Age <40	2(15.4)	11(84.6)	1.00	1.00
40-64	37 (22.16)	130(77.84)	1.94(0.35-10.86)	1.9(0.34-10.75)
‗65	68(33.3)	136(66.7)	7.43(1.4-19.40)	5.43(1.22-12.1)*
Marital status				
Single	2(25)	6(75)	1.00	1.00
Married	78(32.1)	165(67.9)	2.17(1.23-3.8)	2.12(0.24-8.51)
Widowed	19(17.92)	87(82.08)	1.53(0.286-8.15)	0.45(0.13-1.50)
Divorced	8(29.63)	19(70.37)	1.93(0.74-5.05)	0.66(0.22-1.95)
Number of families				
<3	18(19.35)	75(80.65)	1.00	1.00
3-5	72(30.25)	166(69.75)	1.97(.9-4.26)	1.7(0.66-4.38)
>5	17(32.07)	36(67.93)	1.8(1.00-3.24)	1.3(0.64-2.70)
Residence				
Urban	79(43.65)	102(56.35)	6.41(2.94-14.00)	4.84(2.95-7.95)**
Rural	28(13.8)	175(86.2)	1.00	1.00
Educational level				
Unable to read and write	61(21.1)	228(78.9)	0.53(0.15-1.8)	0.5(0.1-2.65)
Primary Secondary and	21(44.4)	24(55.6)	1.75(0.46-6.65)	0.98(0.19-5.20)
Preparatory	12(54.55)	10(45.45)	2.89(0.66-12.57)	2.13 (0.39-11.62)
College	9(56.25)	7(43.75)	2.57(0.54-12.17)	2.0(0.38-11.17)
University	4(33.33)	8(66.67)	1.00	1.00
Occupation				
Civil servant	6 (37.5)	10(62.5)	1.00	1.00
Farmer	36(24.00)	114(76.00)	6.35(1.1-27.27)	5.18(0.99-17.06)
Housewife	28(23.73)	90(76.27)	1.64(0.35-7.76)	2.4 (0.45-12.6)
Merchant	15(46.88)	17(53.12)	3.3(0.54-20.26)	1.95 (0.36-10.15)
Retire	20(36.36)	35(63.64)	1.77(0.37-8.47)	1.14(0.26-5.08)
Unemployment	2(15.38)	11(84.62)	3.14(0.63-15.64)	1.17(0.1-13.53)
Duration of VI				
<5 years	47(19.26)	197(80.74)	1.00	
‗5 years	60(42.86)	80(57.14)	3.14(1.98-4.99)	3.33(1.88-5.89) ^**^
Dietary type				
Vegetarians	53(35.1)	98(64.9)	1.00	1.00
Non-vegetarians	54(23.18)	179(76.82)	1.79(1.14-2.82)	1.01(0.57-2.17)
Current alcohol drinking				
Yes	20(42.55)	27(57.45)	2.13(1.4-3.99)	0.49 (0.22-1.08)
No	87 (25.82)	250(74.18)	1.00	1.00
Diastolic BP				
<90mmhg	51(25.63)	148(74.37)	1.00	1.00
‗90mmhg	5630.27)	129(69.73)	1.26(0.8-1.97)	0.65(0.37-1.14)

***Extremely significant association at p-value <0.001 **Very significant association at p-value <0.01, COR, Crude odds ratio; AOR, Adjusted odds ratio; CI, Confidence interval; VI, visual impairment; DBP, diastolic blood pressure.
